# Onboarding obesity management in cardiovascular care: A cardiologist's guide to latest advances

**DOI:** 10.1016/j.ajpc.2025.100987

**Published:** 2025-04-03

**Authors:** François Schiele, François Dievart, David Jacobi, Denis Angoulvant, Sebastien Czernichow, Etienne Puymirat, Pierre Sabouret, Victor Aboyans

**Affiliations:** a Department of Cardiology, University Hospital Besancon, Besancon, France; b EA3920, SINERGIES University of Franche-Comté, Besancon, France; c Department of Cardiology, Villette Private Hospital, Dunkerque, France; d Institut du Thorax, Nantes University, CHU Nantes, CNRS, and INSERM, Nantes, France; e Department of Cardiology, University Hospital of Tours, Tours, France; f Department of Nutrition, Georges Pompidou European Hospital, Assistance Publique-Hôpitaux de Paris, University Paris Cité, Paris, France; g Department of Cardiology, Georges Pompidou European Hospital, Assistance Publique-Hôpitaux de Paris, Paris, France; h Heart Institute, Pitié-Salpêtrière Hospital, Assistance Publique-Hôpitaux de Paris, Paris, France; i Department of Cardiology, Dupuytren University Hospital, Limoges, France; j EpiMaCT, Inserm1094/IRD270, Limoges University, Limoges, France

**Keywords:** Overweight, Obesity, Cardiovascular disease, Cardiologist, Glucagon-like peptide-1 receptor agonists, GLP-1 receptor agonists

## Abstract

In recent decades, the prevalence of obesity has escalated markedly, becoming a serious epidemic and public health crisis requiring urgent and sustained attention. Obesity is associated with a large number of health conditions, including cardiovascular diseases (CVDs), which contribute to an increase in mortality and overall global health challenge. Despite its high morbidity and mortality, most healthcare practitioners perceive obesity as an outcome of unhealthy lifestyle rather than a disease by itself. As such, obesity is either overlooked or considered a minor risk factor for CVD in clinical practice, among others. Since cardiovascular (CV) causes remain the leading cause of death in patients with obesity, cardiologists are among the most frequently visited healthcare professionals and can play an essential role in addressing this disease. Obesity is a complex, chronic, relapsing yet treatable disease that stems from the disruption in the body's homeostatic, hedonic, and cognitive systems, as a result of an interplay between genetic, metabolic, inflammatory, vascular, environmental and behavioral, and pharmacological factors. With early recognition and assessment, management of this disease can successfully improve life expectancy and reduce CV risk. In this review, a concise overview of obesity was provided, focusing on its pathophysiology, diagnosis, and management. The correlation between obesity and CVDs was further discussed, highlighting the significance of obesity education and management among cardiologists to improve patient outcomes and prevent the progression of obesity and its related comorbidities.

## Central illustration

Obesity disrupts key pathways involved in energy regulation and homeostasis, and its management requires a well-coordinated and effective multidisciplinary approach to prevent associated clinical complications.

**Figure:**
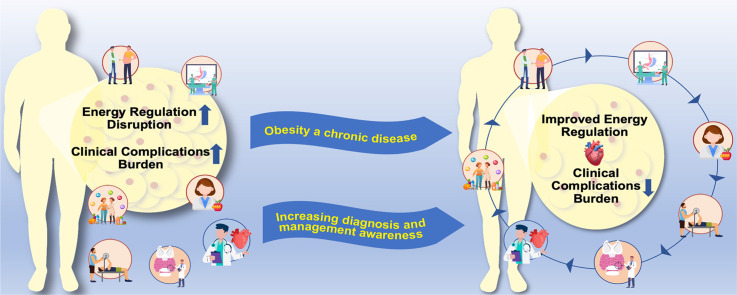

## 1 Introduction

Over the past few decades, the prevalence of obesity has dramatically increased, constituting a real epidemic and global public health crisis involving hundreds of millions of people worldwide [1]. In tandem with this rise of obesity, there has been an escalation in the risk and premature onset of various associated conditions including cardiovascular diseases (CVDs) and type 2 diabetes mellitus (T2DM), which contribute to high morbidity and mortality [1,2].

Most healthcare practitioners view obesity as an outcome of unhealthy lifestyle and eating behaviors and consider it a minor risk factor for CVD among others, rather than a disease by itself [3]. Despite abdominal obesity being acknowledged as a risk factor for atherosclerosis, cardiologists have traditionally perceived obesity differently, often considering it to offer a degree of protection in cases of myocardial infarction (MI) or heart failure (HF), a concept commonly referred to as the "obesity paradox" [4,5]. Nevertheless, it is widely acknowledged that obesity is linked to decreased life expectancy, particularly due to cardiovascular (CV) reasons, which account for two-thirds of cases [6].

This perception among most healthcare practitioners can lead to either a lack of identifying obesity in early stages or proceeding directly to management, without appropriate diagnosis [1,7]. In 2021, the European Commission acknowledged obesity as a definite pathological identity, representing a chronic relapsing non-communicable disease, and highlighted the need for an increased obesity education among healthcare professionals, particularly cardiologists, who are most frequently visited by this patient population [7,8]. In this review, we discuss how cardiologists may enhance their understanding of the interplay between obesity and CVDs, to be part of a comprehensive multidisciplinary team in recognizing and managing obesity early on in clinical practice.

## 2 General overview of obesity

The World Health Organization (WHO) defines obesity as a chronic complex disease of excessive fat deposits, which impairs a person's health. The obesity epidemic started in most developed countries in the 1970s and 1980s, and since then, its worldwide prevalence among children and adults has more than doubled [1,9]. This rise in obesity over the last decades has been driven by several factors, particularly the gradual and global shift in lifestyle [9]. In 2022, an estimated worldwide 2.5 billion adults aged 18 years and older were considered to have excess weight, of whom 890 million adults (36 %) were classified as living with obesity. This marks an increase in the global adult population with excess weight from 25 % in 1990 to 43 % in 2022, significantly impacting morbidity, mortality, and health care expenditures [1].

### 2.1 Interconnected systems at the root of obesity

**Fig. 1:**
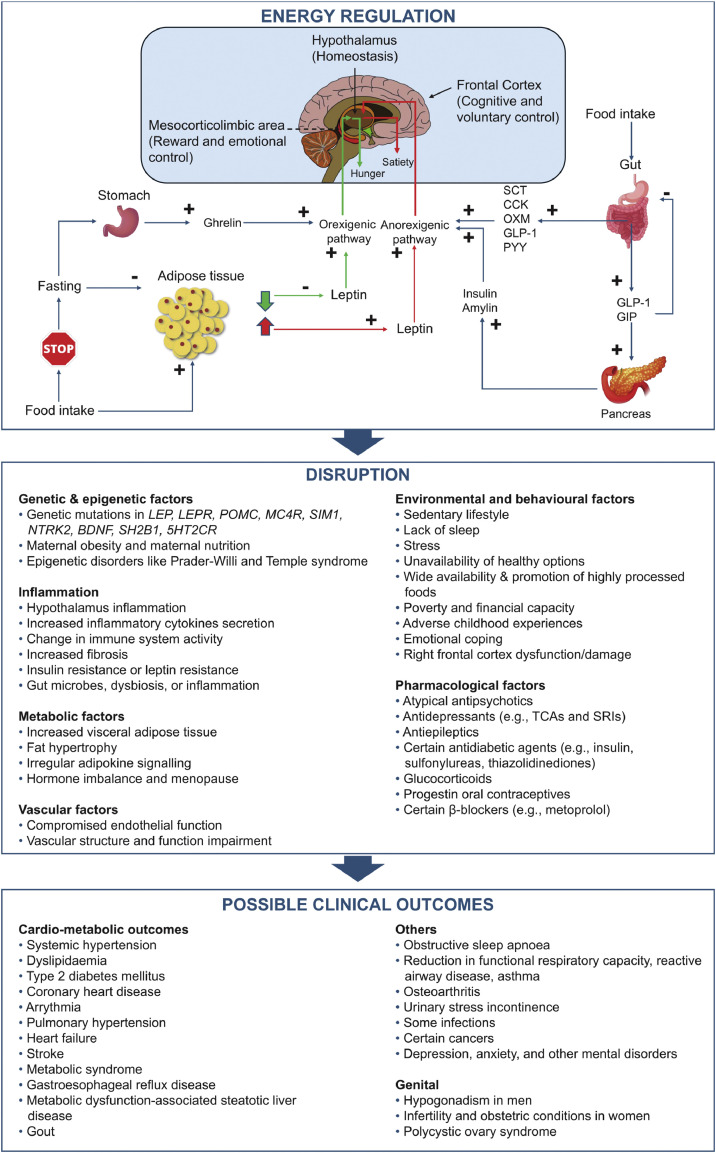
Key players in body energy regulation and disruption factors leading to overweight or obesity and related health outcomes. Abbreviations: BDNF, Brain Derived Neurotropic factor; CCK, cholecystokinin; GIP, glucose-dependent insulinotropic polypeptide; GLP-1, glucagon-like peptide-1; LEP, leptin; LEPR, leptin receptor; MC4R, melanocortin 4 receptor; NTRK2, Neurotrophic Tyrosine Kinase Receptor Type 2; OXM, oxyntomodulin; POMC, proopiomelanocortin; PYY, peptide tyrosine; SCT, secretin; SH2B1, SH2B adaptor protein; 5HT2CR, serotonin (5-HT) 2C receptor; SIM1, Single-minded Drosophila Homologue-1; SRIs: serotonin reuptake inhibitors; TCAs, tricyclic antidepressants. (**+**) represents positive feedback or activation (**-**) represents negative feedback or inhibition.

The human body regulates the amount of adipose tissue over time by balancing caloric consumption and food intake to maintain energy reserves through multiple systems: the homeostatic system, referred to as the “metabolic brain”, the hedonic system, referred to as the “emotional brain”, and the cognitive system (Fig. 1) [10,11].

The primary homeostatic energy system allows the bidirectional communication between the brain, mainly the hypothalamus, and peripheral organs through neuronal, hormonal, or food-derived chemical signals that activate neural circuits. These neural circuits can be orexigenic, which stimulate hunger, or anorexigenic, which leads to satiety following food intake and decreases energy consumption [3,10]. A key circulating mediator of neural circuits is leptin, which is a protein produced primarily by adipose tissue. Hence, serum leptin levels are positively associated with fat mass. Leptin levels increase after meals, suppressing the feeling of hunger and promoting satiety, and decrease during fasting [3,10]. For their part, food-derived chemicals regulate the neural circuits, promoting hunger or satiety. Following food intake, chemicals with satiety-stimulating effects are released, essentially by the duodenum, small intestine, and pancreas. In contrast, during fasting, the hunger-stimulating gastrointestinal hormone, ghrelin, is released from the stomach and activates the orexigenic pathway (Fig. 1) [12].

Interacting with the homeostatic energy system, another important component is the hedonic system in the mesocorticolimbic area, which governs the reward control system through the neurotransmitters serotonin and dopamine [11]. These reward-related brain regions respond to visual cues of desirable foods and reinforce cravings and pursuit of these foods through unconscious learning mechanisms, independent of hunger signals [3]. Increased serotonergic signaling typically results in reduced food intake and weight loss, while an increase in dopaminergic signaling is associated with food overconsumption and weight gain. Lastly, the cognitive system, situated in the prefrontal cortex, plays a significant role in the voluntary control mechanisms, including social and impulse control, which influence food choices, attitude towards eating, and eating frequency [11].

### 2.2 Disruption of homeostasis causing obesity

Obesity is a heterogeneous, complex, and multifactorial chronic disease characterized by both abnormal and/or excess body fat accumulation [13,14]. In general, obesity occurs when a genetically predisposed individual is exposed to an obesogenic environment for a long period of time [10].

The disruption in the three above-described systems, particularly the homeostatic energy system, is key behind the development of obesity [10]. Obesity is influenced by a variety of factors, categorized as genetic and epigenetic, metabolic, inflammatory, vascular, environmental and behavioral, or pharmacological (Fig. 1). Hypothalamic inflammation may be the primary mechanism leading to unwanted weight loss or obesity [15]. An excess in visceral adipose tissue triggers a pathological process mediated by an increased cytokine secretion, variations in immune system activity, fat hypertrophy, increased fibrosis, and alterations in vascular function and structure [3,10,15]. Moreover, studies have reported genetic mutations affecting key proteins in energy homeostasis and genes that can predispose individuals to develop obesity [16]. An example of these mutations is an alteration in leptin structure or leptin receptor (LEPR), leading to the inability of leptin to bind to its specific receptor and contributing to obesity via complex mechanisms that remain not fully understood [3,10]. This disruption results in irregular adipokine signaling, compromised endothelial function, and weight-related comorbidities or conditions [10].

Nevertheless, genetics cannot completely explain the significant surge in obesity cases over the last few decades. Environmental and behavioral factors such as nutrition and lifestyle may also alter gene expression without affecting the DNA sequence, a term known as epigenetics [16]. Likewise, certain clinical conditions or pharmacological agents including some β-blockers (metoprolol) and some antidiabetic agents (insulin, sulfonylurea, and thiazolidinediones), may trigger or contribute to excessive adipose tissue [10,14].

### 2.3 Clinical outcomes of disrupted systems

Obesity is associated with a large number of health conditions, confounding its diagnosis and management. T2DM, dyslipidemia, obstructive sleep apnea (OSA), and metabolic dysfunction-associated steatotic liver disease illustrate some of the weight-related conditions, other than CVD, which are either caused or exacerbated by obesity (Fig. 1) [10].

### 2.4 Diagnosing obesity

**Fig. 2:**
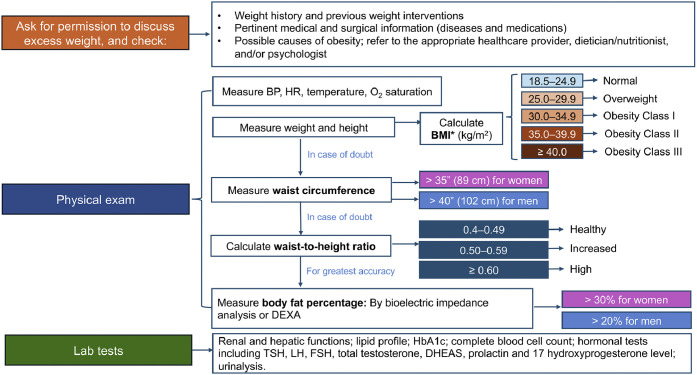
Diagnosis and assessment of obesity in primary care. Abbreviations: BMI, body mass index; BP, blood pressure; DEXA, dual-energy X-ray absorptiometry; DHEAS, dehydroepiandrosterone-sulfate; FSH, follicle stimulating hormone; HbA1c, hemoglobin A1c; HR, hear rate; LH, luteinizing hormone; O2, oxygen; TSH, thyroid stimulating hormone. *BMI cut-offs for non-Asian ethnicity. For Asian ethnicity, patients with a BMI of 23 to 27.4 kg/m^2^ are considered overweight, while patients with a BMI ≥27.5 kg/m^2^ are classified as having obesity. The WHO weight classifications in adult Asians are as follows: BMI <18.5 kg/m^2^ indicates underweight, 18.5 to 22.9 kg/m^2^ normal weight, 23 to 24.9 kg/m^2^ overweight, 25 to 29.9 kg/m^2^ obesity class I, and ≥30 kg/m^2^ obesity class II [17].

The diagnosis of obesity begins by assessing the patient's history, vital signs, and laboratory tests (**Table S1**) and is primarily confirmed by the patient's body mass index (BMI), which is calculated by dividing weight in kilograms by the square of height in meters [1]. The BMI is considered a simple, easy, and inexpensive tool for identifying patients with obesity and is mostly used in literature and clinical practice [3]. In general, patients with a BMI ≥25 to <30 kg/m^2^ are classified as having overweight while patients with a BMI ≥30 kg/m^2^ are classified as having obesity [1,17]. Of note, ethnicity should also be taken into account, as patients from South, Southeast, and East Asia have different cut-offs. Among this population, patients with a BMI of 23 to 27.4 kg/m^2^ are considered as having overweight, while patients with a BMI ≥27.5 kg/m^2^ are classified as having obesity [17]. Based on the BMI range, obesity is further divided into class I, II, and III (Fig. 2).

Even though BMI is well-correlated with prognosis, using BMI alone may be insufficient for diagnosing and staging obesity due to its inability to distinguish body composition, the location of adipose tissue, and the impact of excess adiposity [17]. In general, overall obesity and abdominal obesity are related, but abdominal or visceral fat may be present in excess in patients with BMI <30 kg/m^2^. An increase in visceral adipose tissue beyond a specific level triggers pathological processes mediated by increased cytokine secretion, changes in immune system activity, hypertrophy of adipocytes, increased fibrosis, and impaired vascular structure and function [3]. Compared to total body fat, visceral fat is more strongly correlated with adverse health effects such as increased morbidity, including CVD, metabolic syndrome, and insulin resistance and mortality risk [3,18]. A patient with normal BMI but elevated visceral abdominal fat should be differentiated from a patient with high BMI and normal visceral abdominal fat, resulting in a more accurate CV risk assessment [3]. Central obesity has been incorporated in some clinical classifications such as the Cardiometabolic Disease Staging system, which accounts for metabolic factors and CV events in addition to the waist circumference. With the increase in the Cardiometabolic Disease Staging system stage, the risk of CV mortality increases with hazard ratios ranging from 3.6 for stage 1 to 14.6 for stage 4 [18].

Therefore, when using the BMI to define excess adiposity, the American Association of Clinical Endocrinologists (AACE) and American College of Endocrinology (ACE) guidelines recommend considering age, gender, ethnicity, fluid status, and muscularity in addition to clinical judgement [17]. When a clinician suspects obesity, clinical judgement should be based on other measurements of adiposity that are better predictors of fat distribution and central obesity and have a closer association with morbidity, such as waist circumference (WC), waist-to-hip ratio (WHR), and mainly waist-to-height ratio (WHtR) [13,14,17]. In fact, measuring WC is recommended in all patients with a BMI <35 kg/m^2^ [17]. Body fat percentage can improve obesity characterization and is measured using body composition technologies such as bioelectric impedance analysis (BIA), dual-energy X-ray absorptiometry (DEXA), and for research purposes, air displacement plethysmography (ADP). These technologies, while more accurate, may be limited by availability, cost, patient status, or lack of validated cut-off points. Bioelectric impedance analysis is cheap and accessible, air displacement plethysmography requires expensive apparatus, and DEXA is more suited for research [17]. Similar to ADP, BIA can assess the overall body density and measure total body fat and lean tissue but are unable to determine fat regional distributions. The precision and accuracy of BIA in measuring visceral adipose tissue is considered medium compared to circumferences, skinfold thickness, and ultrasound, which are considered low, ADP and DEXA, which are considered high, and CT and MRI, which are considered the gold standard. In fact, CT and MRI, followed by DEXA, are considered the most accurate and precise techniques in detecting and measuring fat regional distribution, such as visceral, muscle, or liver fat [3]. Due to potential sources of error with BIA measurements, different BIA model parameters are required depending on age, gender, physical activity level, amount of body fat, and ethnicity for it to be reliable [19].

### 2.5 Obesity classification tools

Obesity should be classified not only based on weight but also on the presence of obesity-related risk factors. Due to the high risk of weight-related comorbidities, evaluation should include measurement with adapted cuff of blood pressure (BP) and laboratory tests which comprise, but are not limited to, fasting plasma glucose, glycated hemoglobin (HbA1c), and lipid panel to identify and assess weight-related diseases and complications [17] (**Table S1**). To assist physicians in clinical decision making, the Edmonton Obesity Staging System (EOSS) is a frequently used classification tool to evaluate the presence of weight-related risk factors or comorbidities, physical symptoms, psychological symptoms, and functional limitations [20]. Based on the EOSS, patients are classified into 5 stages: stage 0 (no risk factor), stage 1 (subclinical risk factor), stage 2 (established disease), stage 3 (severe disease), and stage 4 (end stage disease). The EOSS can aid physicians in understanding the patient's health status and associated morbidity or mortality risks [3]. Clinical staging is employed to assess and characterize the patient's health status and progression of chronic diseases. It serves as a representation of disease severity, facilitating prognostic predictions and guiding therapeutic interventions [20].

### 2.6 Management of obesity

Since obesity is multifactorial, a multidisciplinary approach is desirable [13,14]. In recent years, advances have occurred in three modalities of obesity management: lifestyle intervention, pharmacotherapy, and metabolic bariatric surgery (MBS) [17].

The foundation of obesity management is lifestyle intervention, even when pharmacological therapy or surgery are indicated [2]. For all patients who are overweight or living with obesity, a structured lifestyle intervention program consisting of a healthy meal plan, physical activity, and behavioral interventions should be implemented (Table 1). Caloric intake should be reduced and evidence-based Mediterranean diets and ketogenic diets such as the very low-calorie ketogenic (VLCK) diet may be encouraged in some patients based on their preferences and needs, in addition to increased physical activity, aiming for ≥150 min/week of moderate exercise divided into 3 to 5 sessions per week [17,21]. Patients should be encouraged to adhere to the behavioral interventions by self-monitoring their weight, food intake, and physical activity, as well as participating in long-term programs, support groups, and psychological counseling, if needed [17]. Nevertheless, the effectiveness and durability of lifestyle and behavioral interventions are limited [2].

**Table 1:** Lifestyle interventions.

**Intervention**	**Recommendation**
Diet	• Reduction in calorie intake to 1200–1500 kcal/day for women and 1500–1850 kcal/day for men or energy deficit of 500 to 750 kcal/day • Caloric restriction should be tailored to individual patient needs and health conditions • Evidence-based Mediterranean diet, ketogenic diets including the very low-calorie ketogenic (VLCK) diet, and diets that restrict certain food types such as high-carbohydrate foods, low-fiber foods, or high-fat foods • Refer patients to a medical nutritionist and dietician
Exercise	• Reduction of sedentary behavior • Increased resistance exercise and aerobic physical activity, such as brisk walking, for ≥150 min/week divided into 3 to 5 sessions or ≥30 min/day most days of the week • Higher levels of physical activity, approximately 200 to 300 min/week, recommended to maintain lost weight or minimize weight regain in the long term (>1 year) • Refer patients to exercise specialists, health counsellors, or professionals in physical training
Behavior Therapy and Counselling	• Refer patients to comprehensive lifestyle program or electronically delivered weight loss programs for ≥6 months • Include regular self-monitoring of food intake, physical activity, and weight • Refer to psychological counsellor patients with emotional eating and binge eating disorders; depression and anxiety; body image issues and low self-esteem; stress management; coexisting psychiatric disorders; pre- and post-bariatric surgery; and motivation and adherence issues

Adapted from: [17]; [21]; [47].

Several pharmacological therapies have been developed and approved for the long-term management of obesity [2]. The AACE and ACE guidelines and the American Gastroenterological Association (AGA) guidelines recommend adding pharmacological agents to lifestyle interventions in adults with obesity, or those with overweight and weight-related complications, who have a suboptimal clinical response to lifestyle interventions alone [2,17]. The commonly used pharmacological agents are phentermine-topiramate, naltrexone-bupropion, orlistat, and more recently, liraglutide (3 mg), semaglutide (2.4 mg), and tirzepatide (5 mg, 10 mg, or 15 mg); however, the availability of these agents may vary from one country to another [2,17,22]. Setmelanotide has also been approved for the treatment of obesity and hunger due to genetic disorders such as Bardet-Biedl syndrome, pro-opiomelanocortin (POMC) loss-of-function, including proprotein convertase subtilisin/kexin type 1 (PCSK1), and LEPR deficiency [23]. When choosing between these agents, it is important to consider the patient's clinical profile, including comorbidities, preferences, costs, and access to therapy (Table 2) [2,17,22].

**Table 2:** Considerations for the use of pharmacological agents in the management of obesity.

**Pharmacological agent**	**Special consideration**
Semaglutide 2.4 mg	• May be preferred over other approved obesity management medication (OMM) • Has glucoregulatory benefit • May cause transient mild or moderate nausea, constipation, diarrhea, and vomiting – gradual dose titration may mitigate it • Use with caution and monitor patients with a history of diabetic retinopathy • Contraindicated in patients with personal or family history of medullary thyroid carcinoma and in patients with Multiple Endocrine Neoplasia syndrome type 2
Liraglutide 3 mg	• May be preferred over other approved OMM • Has glucoregulatory benefit • May cause nausea, constipation, diarrhea, and vomiting – gradual dose titration may mitigate it • GLP-1 RAs have been associated with increased risk of gallbladder diseases • Contraindicated in patients with personal or family history of medullary thyroid carcinoma and in patients with Multiple Endocrine Neoplasia syndrome type 2
Tirzepatide 5 mg, 10 mg, and 15 mg	• May be preferred over other approved OMM • Has glucoregulatory benefit and is approved for T2DM • May cause nausea, vomiting, constipation, and diarrhea • May cause acute gallbladder disease and acute pancreatitis • Use with caution in patients with a history of pancreatitis or severe gastrointestinal diseases, including gastroparesis, due to limited studies • Use with caution and monitor patients with a history of diabetic retinopathy • Monitor for depression or suicidal thoughts • Contraindicated in patients with personal or family history of medullary thyroid carcinoma and in patients with Multiple Endocrine Neoplasia syndrome type 2
Phentermine-topiramate	• Teratogenic – Women of childbearing potential should use effective contraception • Monitor for depression or suicidal thoughts • Monitor HR, especially in those with cardiac or cerebrovascular disease • Contraindicated in pregnancy and in patients with glaucoma or hyperthyroidism
Naltrexone-bupropion	• Monitor for depression or suicidal thoughts • Monitor BP and HR in all patients, especially those with cardiac or cerebrovascular disease • Contraindicated in patients with uncontrolled systemic hypertension, seizure disorders, or chronic opioid use
Orlistat	• Advise patients to take multivitamins daily (fat-soluble vitamins: A, D, E, K) 2 h apart from orlistat • Contraindicated in pregnancy and in patients with chronic malabsorption syndrome or cholestasis

Adapted from: [2]; [17]; [26]; [27].

Abbreviations: BP, blood pressure; CVD, cardiovascular disease; GLP-1 RA, glucagon-like peptide-1 receptor agonist; HR, heart rate; OMM, obesity management medication; T2DM, type 2 diabetes mellitus.

Pharmacological agents with incretin mimetic effects, such as liraglutide (3 mg), semaglutide (2.4 mg), and tirzepatide (5 mg, 10 mg, and 15 mg), have demonstrated significant weight reduction in clinical trials. Regarding liraglutide, a meta-analysis of five large-scale phase III trials in patients with overweight and obesity demonstrated a consistent weight loss with a greater likelihood of achieving at least 5 % and 10 % weight loss compared to placebo [24]. In the global phase 3 Semaglutide Treatment Effect in People with Obesity (STEP) program, semaglutide 2.4 mg resulted in greater body weight reduction and achievement of weight-loss targets than lifestyle intervention alone in patients with overweight (BMI ≥27 kg/m^2^ with at least one weight-related complication) or obesity (BMI ≥30 kg/m^2^), with or without weight-related complications [25]. In the STEP 1 trial, patients without T2DM who received semaglutide 2.4 mg with lifestyle intervention for 68 weeks had a 14.9 % body weight reduction from baseline compared to a 2.4 % reduction in those who received lifestyle intervention alone [25]. Recently, the FDA and EMA have approved the glucose-lowering agent, tirzepatide, a novel first-in-class glucose-dependent insulinotropic polypeptide (GIP) and glucagon-like peptide-1 (GLP-1) receptor agonist, at a dose of 5 mg, 10 mg, and 15 mg [26,27]. In the SURMOUNT-1 trial, which included patients with overweight and at least one weight-related complication or obesity without T2DM, tirzepatide was associated with a decrease in body weight of 15 %, 19.5 %, and 20.9 % at a dose of 5 mg, 10 mg, and 15 mg, respectively, from baseline to week 72 compared to placebo [22]. Table 3 summarizes promising pharmacological agents and combinations that are currently under investigation for the management of obesity.

**Table 3:** Promising pharmacological agents and combinations under investigation for the management of obesity.

**Pharmacological agent**	**Class**	**Administration route**	**Population**
*Phase III*			
Orforglipron	GLP-1 RA	Oral	Adults with obesity (BMI ≥30 kg/m^2^) or overweight (BMI ≥27 kg/m^2^) and ≥1 wt-related comorbidity without T2DM (**ATTAIN-1**) or with T2DM (**ATTAIN-2**) Adults with T2DM and obesity or overweight with increased CV risk (**ACHIEVE-4**)
CagriSema (cagrilintide + semaglutide)	Amylin analogue + GLP-1 RA	SC	Adults with obesity (BMI ≥30 kg/m^2^) or overweight (BMI ≥27 kg/m^2^) and ≥1 wt-related comorbidity without T2DM (**REDEFINE 1**) Adults with BMI ≥27 kg/m^2^ with T2DM (**REDEFINE 2**) Adults with established CVD (**REDEFINE 3**)
Survodutide	Dual glucagon receptor agonist and GLP-1 RA	SC	Adults with obesity (BMI ≥30 kg/m^2^) or overweight (BMI ≥27 kg/m^2^) and ≥1 wt-related comorbidity without T2DM (**SYNCHRONIZE-1**) or with T2DM (**SYNCHRONIZE-2**) or with established CVD (**SYNCHRONIZE - CVOT**)
Mazdutide	GIPR agonist and glucagon receptor agonist	SC	Adults with obesity (BMI ≥28 kg/m^2^) or overweight (BMI ≥24 kg/m^2^) and ≥1 wt-related comorbidity without T2DM (**GLORY-1**)
Retatrutide	GIPR agonist, glucagon receptor agonist, and GLP-1 RA	SC	Adults with obesity (BMI ≥30 kg/m^2^) or overweight (BMI ≥27 kg/m^2^) and ≥1 wt-related comorbidity without T2DM (**TRIUMPH-1**)
*Phase II*			
Cagrilintide	Amylin receptor agonist	SC	Adults with obesity (BMI ≥30 kg/m^2^) or overweight (BMI ≥27 kg/m^2^) and hypertension or dyslipidemia without T2DM
PYY 1875	PYY RA	SC	Adults with BMI of 25 to 35 kg/m^2^ without T2DM
Efinopegdutide	Dual glucagon receptor agonist and GLP-1 RA	SC	Adults with severe obesity (BMI≥35 to ≤50 kg/m^2^) without T2DM
Pemvidutide	Dual glucagon receptor agonist and GLP-1 RA	SC	Adults with obesity (BMI ≥30 kg/m^2^) or overweight (BMI ≥27 kg/m^2^) and ≥1 wt-related comorbidity without T2DM
Maridebart cafraglutide (AMG-133/MariTide)	GIPR antagonist and GLP-1 RA	SC	Adults with obesity (BMI ≥30 kg/m^2^) or overweight (BMI ≥27 kg/m^2^) and ≥1 wt-related comorbidity with or without T2DM
NNC0165–1875 + Semaglutide	GLP-1 RA + PYY RA	SC	Adults with BMI of 30 to 45 kg/m^2^ without T2DM
Dapiglutide	GLP-1 RA + GLP-2 RA	SC	Adults with BMI ≥30 kg/m^2^ without T2DM
Semaglutide + bimagrumab	GLP-1 RA + Activin type II receptor blocking biologic	Semaglutide: SC Bimagrumab: IV	Adults with obesity (BMI ≥30 kg/m^2^) or overweight (BMI ≥27 kg/m^2^) and ≥1 wt-related comorbidity without T2DM
S-309,309	MGAT2	Oral	Adults with obesity (BMI ≥30 kg/m^2^) without T2DM

Abbreviations: BMI, body mass index; CV, cardiovascular; CVD, cardiovascular disease; GIPR, Glucose-dependent insulinotropic peptide receptor; GLP-1 RA, glucagon-like peptide-1 receptor agonist; GLP-2 RA, glucagon-like peptide-2 receptor agonist; IV, intravenous; MGAT2, Monoacylglyceroltransferase; PYY, peptide YY; SC, subcutaneous; T2DM, type 2 diabetes mellitus.

Metabolic bariatric surgery can be a highly effective and durable treatment for weight loss and obesity-related comorbidities in eligible patients [28]. It is considered the most effective treatment for obesity across all BMI classes due to its effects in reducing weight, improving comorbid conditions, and enhancing overall quality of life (QoL). Moreover, some observational data and non-blinded randomised trials suggest that bariatric surgery could decrease mortality [28,29]. In clinical practice, the most used approaches are sleeve gastrectomy and Roux-en-Y gastric bypass [29]. Metabolic bariatric surgery is recommended for patients with a BMI ≥35 kg/m^2^ regardless of the presence of weight-related comorbidities and considered for those with a BMI of 30 kg/m^2^ to 34.9 kg/m^2^, who have not achieved substantial and durable weight loss or improvement in comorbidities using nonsurgical methods [28]. Another interventional method is the endoscopic metabolic and bariatric therapy, mainly intragastric balloon and endoscopic sleeve gastroplasty (ESG), which is indicated for patients with a BMI of 30 kg/m^2^ to 40 kg/m^2^ or a BMI >27 kg/m^2^ with at least one obesity-related comorbidity. Recently, the FDA has approved ESG for a BMI up to 50 kg/m^2^ [29].

Since gender-related differences in response to obesity treatment have not been extensively studied, current therapeutic management are not gender-adjusted. Men and women achieve similar weight loss levels following bariatric surgery. Nevertheless, women are more likely to require corrective procedures and experience poorer psychological outcomes, whereas men tend to experience worse physiological outcomes and less improvement in comorbidities, such as hyperlipidemia, insulin-dependent diabetes, and sleep apnea [30]. Beyond bariatric surgery, gender differences in obesity management outcomes also extend to non-pharmacological and pharmacological interventions. While non-pharmacological interventions appear slightly more effective in men than women, the long-term differences are negligible. Gender representation in weight management clinical trials remains imbalanced, with men typically underrepresented despite similar weight loss outcomes between genders. This underrepresentation makes it challenging to fully assess potential differences in treatment responses. Notably, among available data, women experience greater weight loss than men after 1 year of pharmacotherapy, such as with semaglutide or liraglutide, though they are more likely to encounter gastrointestinal adverse events [31].

## 3 Embracing the management of obesity in cardiology

### 3.1 Obesity and cardiovascular disease interplay

Patients with overweight and obesity are at an increased risk of CV morbidity and mortality. Multiple epidemiological studies highlight a non-linear relationship between BMI and all-cause mortality risk [13,14]. An analysis including patients from the “Helseundersøkelsen i Nord-Trøndelag” (HUNT) Norwegian study and the UK Biobank cohort found that an increase in BMI by 1 unit was associated with a 5 % and 9 % higher risk of mortality in patients with overweight and obesity, respectively [32]. Specifically, patients with a BMI ≥25 kg/m^2^ face a significantly increased risk of death from CVD, in particular coronary heart disease and ischemic stroke [33].

Obesity is linked to many CVDs [13,14,34]. Although the pathogenesis behind CVD morbidity and mortality in obesity was previously thought to be solely indirect, through the increase in conventional CV risk factors and comorbidities, emerging evidence highlight the presence of a direct causal relationship between obesity and CVD as well [13,14].

Undoubtedly, obesity leads to the development of coronary artery disease [35]. Excess adiposity, particularly visceral adiposity, accelerates the progression of atherosclerosis within the coronary arteries and aorta and is linked to abnormalities in the coronary microvasculature, which is a key regulator of coronary flow reserve [36]. Likewise, obesity increases the risk of HF through obesity-related hemodynamic and anatomic cardiac changes, in addition to other metabolic, inflammatory, and hormonal changes. Altered myocardial metabolism affects fatty acid and glucose oxidation and results in abnormal cardiac substrate utilization, impaired cardiac efficiency, and decreased energy generation, all of which produce the functional consequences of HF, especially HF with preserved ejection fraction (pEF) [36]. In the Framingham Heart Study, a BMI increase by 1 unit led to a 5 % increase in HF risk in men and 7 % increase in women after adjusting for established risk factors [37].

Excess adiposity also increases the risk of developing atrial fibrillation (AF) and its progression from paroxysmal to more permanent AF, thereby increasing morbidity and mortality. This may be attributed to several mechanisms such as atrial structural and electrical remodeling, which contribute to the development of the arrhythmogenic substrate [36]. During the follow-up in the Framingham Heart Study, each 1-unit increase in BMI was found to increase the risk of new-onset AF by 4 % [38]. Similarly, patients with obesity have a higher risk of ventricular tachycardia or fibrillation. The association between obesity and ventricular dysrhythmia is potentially linked to an increase in cardiac electrical irritability, abnormal late potentials, and disrupted sympathovagal balance, leading to more frequent and complex ventricular dysrhythmias, even in the absence of clinically overt HF [36]. Additionally, studies have found an association between obesity and pulmonary hypertension. In a large hospital-based sample undergoing invasive hemodynamic measurements, the prevalence of pulmonary hypertension was found to increase from 51 % in patients without obesity to 79 % in patients with class III obesity [34]. The link between obesity and pulmonary hypertension seems independent of obesity-related conditions such as OSA, and may be due to obesity-related metabolic dysfunction and pulmonary vascular remodeling [34].

### 3.2 Advancing cardiometabolic care: obesity management

Studies have shown that weight loss can lead to improvements in CVD risk factors and adverse CV events. Table 4 summarizes key trials on weight management and CV benefits. The Look AHEAD trial highlighted the role of lifestyle intervention in reducing CV outcomes [39]. Furthermore, in long-term studies, such as the SOS trial, MBS was associated with improvements in mortality and CV risk factors and outcomes [40,41]. Obesity management medications, particularly semaglutide, have also been studied in patients with obesity and CVDs. In the SELECT double-blind trial against placebo, semaglutide led to a 20 % reduction in death from CV causes, non-fatal MI, or non-fatal stroke and an 8.15 % reduction in body weight. In addition, the STEP HFpEF study demonstrated semaglutide improved Kansas City Cardiomyopathy Questionnaire clinical summary score (KCCQ-CSS) by 7.8 points, increased 6-minute walking distance by 20.3 m, and reduced body weight by 10.7 % compared to placebo [[42], [43], [44]]. Currently, the SURMOUNT-MMO trial of tirzepatide is underway to investigate its CV effects in adults with obesity and CVD. A few real-world retrospective data on CV outcomes in patients with obesity are currently available. A retrospective study of 85,044 patients with obesity from the US Medicare database found that semaglutide and tirzepatide led to a reduction in CVD outcomes, particularly HF, AF, arrhythmia, and peripheral vascular disease [45]. In a meta-analysis of 10 trials including patients with AF undergoing catheter ablation, weight loss was associated with a greater reduction in recurrent AF at >12 months after ablation compared to placebo, defined as no weight loss. This benefit was consistently seen with both >10 % and <10 % weight loss [46].

**Table 4:** Summary of key trials assessing obesity management and CV outcomes.

**Trial(s)**	**Reference**	**Study description**	**Follow-up**	**Main findings**
Look AHEAD	Look AHEAD Research Group et al. (2013) [39]	Randomized controlled trial involving 5145 adults (45 to 76 years old; 59 % women vs. 41 % men) with T2DM and BMI ≥25 kg/m^2^ (≥27 kg/m^2^ in patients taking insulin), who received lifestyle intervention (decreased calorie intake and increased physical activity) vs. control (diabetes support and education)	Median of 9.6 years	Lifestyle intervention was associated with an initial weight loss of 8.6 % followed by a regain with no reduction in CV outcomes compared to controls after 9.6 years but was terminated and considered futile.
	Look AHEAD Research Group et al. (2016) [48]			Post-hoc analysis of this trial showed that a loss of ≥10 % of body weight during the first year was associated with 21 % reduction in risk of major CV events and 24 % reduction in the risk of secondary CVD outcome relative to the control group.
SOS (MBS)	Sjostrom et al. (2007) [49]	Prospective, controlled trial involving 4047 adults (37 to 60 years old; 71 % women vs. 29 % men) with a BMI ≥34 kg/m^2^ in men and ≥38 kg/m^2^ in women, who received MBS vs. matching conventional nonsurgical treatment	Median of 10.9 years	During the follow-up period of up to 16 years, patients who underwent MBS had a lower overall mortality rate compared to the control group after adjusting for baseline characteristics (adjusted HR, 0.71; 95 % CI, 0.54 to 0.92; *p* = 0.01).
	Sjostrom et al. (2012) [41]		Median of 14.7 years	MBS was associated with a lower incidence of CV deaths (adjusted HR, 0.47; 95 % CI, 0.29 to 0.76; *p* = 0.002), and total CV events (adjusted HR, 0.67; 95 % CI, 0.54 to 0.83; *p* < 0.001) than nonsurgical treatment.
	Sjostrom et al. (2004) [40]		Median of 2 and 10 years	- The incidences of new cases of hypertriglyceridemia, T2DM, and hyperuricemia, which were significantly higher in the MBS group than in the control group. - Remission from arterial hypertension, T2DM, hypertriglyceridemia, low HDL cholesterol level, and hyperuricemia were more common in MBS group than in control group.
SELECT	Lincoff et al. (2023) [43]	Double-blind, placebo-controlled trial involving 17,604 adults (≥45 years old; 72 % men vs. 28 % women) with a BMI ≥27 kg/m^2^ and pre-existing CVD without T2DM, who received once-weekly semaglutide 2.4 mg vs. matching placebo	Mean of 39.8 ± 9.4 months	- Compared to placebo, semaglutide 2.4 mg resulted in a significant reduction in the primary CV event-driven endpoint, defined as death from CV causes, nonfatal MI, or nonfatal stroke (HR, 0.80; 95 % CI, 0.72 to 0.90; *p* < 0.001). - The secondary endpoint of mortality from CV causes was also reduced (HR, 0.85; 95 % CI, 0.71 to 1.01; *p* = 0.07). - In the full analysis population, body weight decreased by 9.39 % from baseline to week 104 in the semaglutide 2.4 mg group and by 0.88 % in the placebo group. - Progression of coronary artery disease was also prevented with a reduction in non-fatal MI (HR 0.72, 0.61 to 0.85) and coronary revascularizations (HR 0.77, 0.68 to 0.87).
	Ryan et al. (2024) [44]		208 weeks	- Semaglutide 2.4 mg resulted in a reduction of 8.7 % in body weight (95 % CI, −11.0 to −9.42; *p* < 0.0001), 6.4 cm in waist circumference (95 % CI, −7.18 to −5.61; *p* < 0.0001), and 5.87 % in waist-to-height ratio (95 % CI, −6.56 to −5.17; *p* < 0.0001) compared to placebo.
STEP-HFpEF	Kosiborod et al. (2023) [42]	Randomized, double-blind, placebo-controlled trial involving 529 adults (≥18 years old; 56 % women vs. 44 % men) with HFpEF (EF ≥45 %) and BMI ≥30 kg/m^2^, who received semaglutide 2.4 mg or matching placebo	52 weeks	- KCCQ-CSS decreased from baseline by 16.6 points with semaglutide 2.4 mg versus 8.7 points with placebo (95 % CI, 4.8 to 10.9; *p* < 0.001), and mean body weight was reduced by 13.3 % with semaglutide 2.4 mg and 2.6 % with placebo (95 % CI, −11.9 to −9.4; *p* < 0.001) in full analysis population. - Greater improvements in 6MWD were noted in patients receiving semaglutide 2.4 mg than on placebo (estimated difference, 20.3 m; 95 % CI, 8.6 to 32.1; *p* < 0.001).
SURMOUNT-MMO	NCT05556512	Double-blind, placebo-controlled trial involving 17,604 adults (≥40 years old) with BMI ≥27 kg/m² and established CVD, receiving tirzepatide up to a maximum tolerated dose or matching placebo	Up to 5 years	Not completed yet* – Primary endpoint will be the time to first occurrence of all-cause death, nonfatal MI, nonfatal stroke, coronary revascularization, or HF events.

Abbreviations: 6MWD, 6-minute walking distance; AF, atrial fibrillation; AHEAD, Action for Health in Diabetes; BMI, body mass index; CI, confidence interval; CVD, cardiovascular disease; EF, ejection fraction; HDL, high-density lipoprotein; HF, heart failure; HFpEF; heart failure with preserved ejection fraction; HR, hazard ratio; KCCQ-CSS, Kansas City Cardiomyopathy Questionnaire clinical summary score; MBS, metabolic bariatric surgery; MI, myocardial infarction; SELECT, Semaglutide Effects on Heart Disease and Stroke in Patients with Overweight or Obesity; SOS, Swedish Obese Subjects; STEP; Semaglutide Treatment Effect in People with Obesity; SURMOUNT-MMO, A Study of Tirzepatide (LY3298176) on the Reduction on Morbidity and Mortality in Adults With Obesity; T2DM, type 2 diabetes mellitus.

*Not completed at the time of manuscript writing.

## 4 Cardiologists' perspective on obesity management

Although studies have shown that higher BMI values are associated with an increase in numerous diseases and CVD risk, a controversy in cardiovascular medicine, known as “obesity paradox”, exists. This paradox correlates a better outcome and improved survival in patients with established CVD and overweight or first-degree obesity (BMI ≥30 kg/m^2^ and <35 kg/m^2^), compared to those who have a BMI <25 kg/m^2^ [3,36]. Since the discovery of this counterintuitive finding, this topic has been debated and studied without any clear interpretation to date. The potential methodological explanations include lack of power, as this paradox was not observed in a larger study, selection bias, misclassification bias caused by using BMI instead of body fat distribution, unmeasured confounding factors such as cardiorespiratory fitness, sarcopenia, smoking, and potential for bias due to illness-related weight loss [33,36]. While the obesity paradox highlights complex interactions between body weight, health, and disease, it does not negate the well-established risks associated with obesity.

Despite the high prevalence of overweight and obesity among people with coronary heart disease, some cardiologists still do not prioritize addressing these conditions with their patients [8]. Indeed, the discussion and treatment of obesity in clinical practice remain limited by the physicians’ lack of time, a belief that the patient's obesity management skills are inadequate, and an inability to recognize obesity as a chronic treatable disease. Raising awareness on treatment evidence may alter these trends and encourage cardiologists to adopt more effective obesity management strategies. For instance, a modest weight loss of 5 to 10 %, even without reaching a normal BMI, can significantly improve CV and metabolic factors by improving glycemia, BP, triglycerides, cholesterol, and QoL and function [8]. Encouraging lifestyle changes remains crucial, even in patients receiving pharmacotherapy. Although the Look AHEAD trial was associated with an initial weight loss of 8.6 % with lifestyle intervention followed by a regain with no reduction in CV outcomes compared to controls after 9.6 years, it was terminated and considered futile [39]. On the contrary, a post-hoc analysis of this trial showed that a loss of ≥10 % of body weight during the first year was associated with a 21 % reduction in the risk of major CV events and 24 % reduction in the risk of secondary CVD outcome relative to the control group [47]. Some explanations proposed by the Look AHEAD trial authors for these results may be the study's lack of sufficient power and the need for greater weight loss (≥10 %) to note an improvement in CV outcomes [8,39].

As the prevalence of obesity increases, physicians, including cardiologists, are likely to encounter more patients with obesity in their clinical practice. Alongside other healthcare professionals, cardiologists play an important role in addressing this epidemic and are encouraged to improve their understanding of obesity to relate to patients affected by such condition, learn how to approach it in clinical practice, and identify patients who are at higher risk of poorer outcomes. Further validation of biomarkers and imaging may enhance the clinical tools to optimize the management of CVD associated with obesity.

Given the complexity of obesity especially in the presence of CVD, it is essential for cardiologists to adopt a multidisciplinary approach including obesiologists and healthcare professionals from various related specialties such as bariatric surgeons, primary care physicians, endocrinologists, medical nutritionists, specialists in physical activity, dieticians, psychologists, and nurses [13,14]. Indeed, despite the diverse management strategies offered by each healthcare provider, effective obesity management necessitates a multidisciplinary approach.

## 5 Conclusion

Obesity is a complex, chronic, and relapsing disease, which has been significantly increasing worldwide over the years and becoming a critical public health crisis requiring urgent and sustained attention. Patients with obesity have a high probability of developing risk factors for CVD and require long-term medical management and multimodal care strategies. Since cardiologists frequently encounter these patients, they are encouraged to develop the necessary knowledge to relate to patients affected by obesity and address this condition in their clinical practice. The possibility of improving patient's outcomes and halting the progression of obesity and its complications will depend on management by a multidisciplinary team and a close collaboration among all involved healthcare professionals, including cardiologists.

## Funding

This work was funded by Novo Nordisk SAS (Paris, France).

## CRediT authorship contribution statement

**François Schiele:** Writing – review & editing, Writing – original draft, Conceptualization. **François Dievart:** Writing – review & editing, Writing – original draft, Conceptualization. **David Jacobi:** Writing – review & editing, Writing – original draft, Conceptualization. **Denis Angoulvant:** Writing – review & editing, Writing – original draft, Conceptualization. **Sebastien Czernichow:** Writing – review & editing, Writing – original draft, Conceptualization. **Etienne Puymirat:** Writing – review & editing, Writing – original draft, Conceptualization. **Pierre Sabouret:** Writing – review & editing, Writing – original draft, Conceptualization. **Victor Aboyans:** Writing – review & editing, Writing – original draft, Conceptualization.

## Declaration of competing interest

The authors declare the following financial interests/personal relationships which may be considered as potential competing interests: Francois Schiele reports article publishing charges and writing assistance were provided by Novo Nordisk Pharma SAS. Overall disclosure is provided within the title page of the manuscript. If there are other authors, they declare that they have no known competing financial interests or personal relationships that could have appeared to influence the work reported in this paper.

## Appendix Supplementary materials

Image, application 1
