# Supplementary material for: Onboarding obesity management in cardiovascular care: A cardiologist's guide to latest advances
Source: Am J Prev Cardiol. 2025 Apr 3;22:100987. doi: 10.1016/j.ajpc.2025.100987 (PMC12035917; doi:10.1016/j.ajpc.2025.100987)
Supplement: Supplementary file 1 [file mmc1.docx]

**Supplementary Material**

**Onboarding Obesity Management in Cardiovascular Care: A Cardiologist’s Guide to Latest Advances**

**Table S1. Laboratory tests in patients with obesity**

| **Laboratory tests** |
| --- |
| ﻿Renal function tests (creatinine, eGFR), electrolytes (sodium, potassium, magnesium, calcium) |
| HbA1c, fasting blood glucose |
| Total cholesterol, HDL- and LDL-cholesterol, triglycerides |
| Alanine aminotransferase and aspartate aminotransferase |
| Complete blood cell count |
| Thyroid stimulating hormone |
| Uric acid |
| ﻿Serum ferritin |
| Vitamins B12 and D levels |
| Urinalysis |
| Urine for micro-proteinuria |
| Women with suspected polycystic ovary syndrome: LH, FSH, total testosterone, DHEAS, prolactin and 17 hydroxyprogesterone level |

Abbreviations: DHEAS, dehydroepiandrosterone; eGFR, estimated glomerular filtration rate; FSH, follicle stimulating hormone; HbA1c, hemoglobin A1c; HDL, high density lipoprotein; LDL, low density lipoprotein; LH, luteinizing hormone.

Reference: [3].
